# Significance of Heparin-Binding Protein and D-dimers in the Early Diagnosis of Spontaneous Bacterial Peritonitis

**DOI:** 10.1155/2018/1969108

**Published:** 2018-09-30

**Authors:** Tomasz Mikuła, Mariusz Sapuła, Joanna Jabłońska, Joanna Kozłowska, Wojciech Stańczak, Dagny Krankowska, Alicja Wiercińska-Drapało

**Affiliations:** Department of Infectious and Tropical Diseases and Hepatology, Medical University of Warsaw, Poland

## Abstract

**Objectives:**

Ascites and spontaneous bacterial peritonitis (SBP) are among the most important complications of decompensated liver cirrhosis. In clinical practice, new inflammation biomarkers are needed for the early diagnosis of SBP, as well-known biomarkers, such as C-reactive protein (CRP), procalcitonin (PCT), or peripheral blood white blood cell (WBC) count, lack the required specificity and sensitivity. The aim of the study was to evaluate the significance of heparin-binding protein (HBP) in comparison to CRP, PCT, WBC, and D-dimers in the diagnosis of SBP.

**Design:**

Cross-sectional descriptive single-center study.

**Setting:**

Department of Infectious and Tropical Diseases and Hepatology, Medical University of Warsaw, Poland.

**Patients:**

All patients admitted to the aforementioned department with decompensated liver cirrhosis and ascites between February 1, 2016, and June 30, 2017.

**Intervention:**

Several markers (HBP, CRP, PCT, WBC, and D-dimers) were analysed in blood serum in regard to their potential use in the diagnosis of SBP in patients with decompensated liver cirrhosis and ascites. We correlated the levels of the aforementioned markers with an ascitic fluid polymorphonuclear count using simple linear regression and multiple linear regression. Sensitivities, specificities, and positive and negative predictive values for SBP were calculated for the aforementioned makers of inflammation.

**Measurements and Main Results:**

A total of 63 patients with decompensated liver cirrhosis and ascites participated in the study. The etiology of liver cirrhosis was varied (HCV: *n* = 40, HBV: *n* = 13, HCV/HBV: *n* = 4, AIH: *n* = 3, PBC: *n* = 2, and haemochromatosis: *n* = 1). After the peritoneal tap, 31 patients were determined to have SBP (defined as an ascitic fluid polymorphonuclear count > 250 cells/*μ*L) and 32 patients had no evidence of SBP on peritoneal tap. A very weak, but statistically significant, correlation of HBP, WBC, and D-dimer levels with the peritoneal fluid polymorphonuclear (PMN) count was observed in the simple regression model, but multivariable analysis using the multiple regression model showed that only D-dimers correlated with peritoneal fluid PMNs independently from other inflammation biomarkers. A D-dimer cutoff value of 1500 ng/mL was determined optimal for ruling out SBP due to high sensitivity (96.8%) and a high negative predictive value (92.9%), although predictably, this marker was not useful for confirming SBP due to low specificity (40.6%) and a low positive predictive value (61.2%). The usefulness of D-dimers was limited by the fact that only 22.2% of the studied patients had D-dimer levels below 1500 ng/mL. HBP and WBC showed little to no predictive value in this study.

**Conclusions:**

D-dimers < 1500 ng/mL make the diagnosis of SBP unlikely, although the peritoneal tap is still the reference method in such situations. In the studied group, the determination of HBP was of no diagnostic benefit in the diagnosis of SBP.

## 1. Introduction

Early detection and optimal treatment of infections remain a common clinical challenge and allow a decrease in the probability of progression to sepsis, septic shock, multiorgan failure, and/or death. Novel laboratory tests for the differentiation of bacterial infection from other types of disease are still in high demand. In everyday clinical practice, biomarkers such as C-reactive protein (CRP), procalcitonin (PCT), or peripheral white blood cell (WBC) count are often used. The levels of these parameters often correlate with biomarkers of coagulation disorders, such as peripheral blood platelet (PLT) count or D-dimers. D-dimers are a biomarker of disseminated intravascular coagulation (DIC) but are also increased in inflammation, thrombosis, or neoplastic disorders. These laboratory tests differ in regard to their sensitivity and specificity, and their values are affected by many factors, e.g., the etiology of the infection, comorbidities, or the age of the patient.

One of the more novel biomarkers of inflammation is heparin-binding protein (HBP). This biomarker is secreted from vesicles of activated neutrophils upon their contact with the endothelium and is internalized by endothelial cells, protecting them from apoptosis. In the infection site, HBP is also secreted during phagocytosis and is responsible for the activation of monocytes in order to prolong their survival and increase the production of cytokines [1, 2].

Increased serum HBP levels have been linked to an increased risk of multiorgan failure and increased mortality in sepsis [3, 4]. In a prospective study conducted by Linder et al., which included 233 adults with fever who were suspected of sepsis, HBP showed a sensitivity in the diagnosis of severe sepsis of 87.1% and a specificity of 95.1%. In that particular study, HBP proved to be the best predictor of severe sepsis [5]. Similar observations in regard to HBP have been made in patients with pneumonia [6, 7].

Patients with liver cirrhosis are immunocompromised, and infections in this population are associated with an increased risk of poor outcomes. What is more, the progression of inflammation can be faster than in immunocompetent patients. The incidence of disorders such as spontaneous bacterial peritonitis (SBP), urinary tract infections, pneumonia, and tuberculosis is about ten times higher than in patients without cirrhosis. The mortality due to infectious diseases in this population is about 20 times higher than that in patients without liver fibrosis. Frequently, the clinical manifestations are much more insidious than in other groups of patients. Some of the reasons for this are long-term disorders of immunity or homeostasis, hypercatabolism, and malnutrition [8–10]. In patients with portal hypertension and hypoalbuminaemia, increased levels of bacterial toxins were found in blood serum, especially in those with oedema of the lower extremities and ascites [11]. Many of these patients have leukopenia and thrombocytopenia, which makes infection prevention even more difficult in these patients. On the one hand, the increased bleeding tendencies are caused by a decreased platelet count and by decreased levels of hepatic, vitamin K-dependent clotting factors. On the other hand, there is an increased risk of vein thrombosis, with 4.5% of adults with liver cirrhosis suffering from portal vein thrombosis. The reasons for this that have been suggested are the decreased blood flow through the portal vein and acquired disorders of clotting factors [12].

Patients with liver cirrhosis have increased concentrations of proinflammatory cytokines such as TNF-*α*, IL-1, and IL-6. A significant increase in IgG, IgA, and IgE immunoglobulin levels in these patients is associated with decreased CD4+ cell counts [13, 14].

One of the complications of ascites in patients with decompensated liver cirrhosis is spontaneous bacterial peritonitis (SBP), which manifests as fever, shivering, pain on abdominal palpation, and feeling unwell, but can be asymptomatic in up to 30% of patients [15].

The confirmation of SBP requires a peritoneal tap with peritoneal fluid analysis and the demonstration of a polymorphonuclear (PMN, neutrophil) count of 250 cells/*μ*L or more. Classically, the main cause of SBP is Gram-negative and/or aerobic bacteria, but in some countries, anaerobic bacteria predominate, and in Europe, the microbiological spectrum of SBP has shifted to Gram-positive bacteria (48%–62%) [16, 17].

In this study, we aimed to determine the significance of HBP in comparison to CRP, PCT, WBC, and D-dimers in the diagnosis of SBP.

## 2. Materials and Methods

The study included patients hospitalised due to decompensated liver cirrhosis and ascites in the Department of Infectious and Tropical Diseases and Hepatology of the Medical University of Warsaw between February 1, 2016, and June 30, 2017.

The patients had their blood samples taken, and some of them were administered antibiotics and subsequently had a peritoneal tap in the first 24 h of the hospitalisation (see Results for a detailed analysis). The blood samples were centrifuged, and the resulting serum was divided into two portions, one of which was used to determine the concentrations of procalcitonin (PCT), C-reactive protein (CRP), and D-dimers, and the other portion was stored at −70°C and later used to determine the heparin-binding protein concentration via the Heparin Binding Protein EIA by Axis-Shield. The peripheral white blood cell (WBC) count and the platelet count (PLT) were determined at hospital admission as well.

All patients signed a written informed consent form, and the study has received the full ethical approval of the Bioethical Committee of the Medical University of Warsaw.

## 3. Statistical Analysis

For data analysis, first we used a simple linear regression model to determine the correlation between age, HBP, plasma D-dimers, WBC, PLT, CRP, and PCT as potential predictors of the peritoneal fluid neutrophil (PMN) count. Independently from the simple linear regression model, we used the multiple regression linear model, in which we initially included the gender, age, HBP, plasma D-dimers, WBC, PLT, CRP, and PCT as potential predictors of the PMN count. A stepwise, backward elimination method guided by the *p* value was used to determine which predictor yielded a statistically significant contribution to the model.

The sensitivities, specificities, and positive and negative predictive values were determined for different cutoff points for select biomarkers.

The Statistica StatSoft package and R were used for statistical analyses.

## 4. Results

A total of 63 patients took part in the study (12 women and 51 men). The etiologies of liver cirrhosis were varied: HCV—40 patients, HBV—13 patients, HCV + HBV coinfection—4 patients, autoimmune hepatitis (AIH)—3 patients, primary biliary cirrhosis (PBC)—2 patients, and haemochromatosis—1 patient; 20 patients (31.7%) were on antibiotic therapy prior to the peritoneal tap (some even before the hospitalisation), with a mean time of antibiotic therapy prior to admission/peritoneal tap of 2.9 days. In our study, SBP was defined as having ≥250 neutrophils/*μ*L in the ascitic fluid acquired during the peritoneal tap. The baseline characteristics of our sample are shown in Table 1.

**Figure 1:**
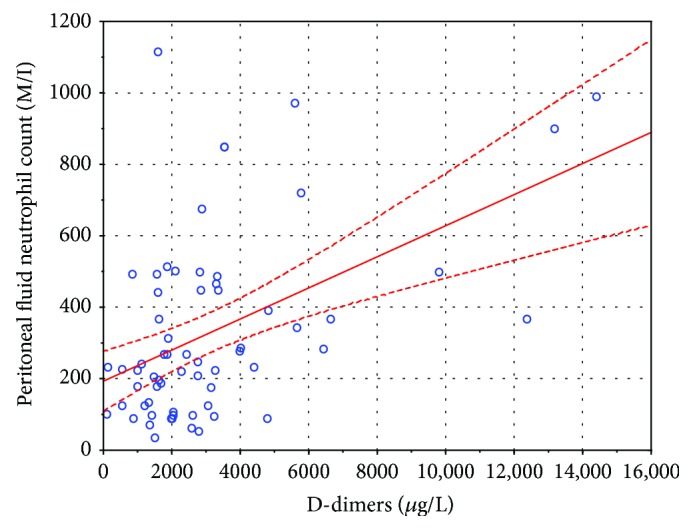
A scatterplot of D-dimers and the PMN count. The regression line is shown by a continuous line, and 95% confidence intervals are shown as a dashed line.

Out of all the aforementioned potential predictors, only plasma D-dimers showed an independent, statistically significant positive correlation with the peritoneal fluid neutrophil count, being the only statistically significant predictor in the multiple linear regression model—see Table 1 and Figure 1.

Both WBC and HBP showed a positive, statistically significant correlation with the peripheral fluid neutrophil count, but only in the simple linear regression model (see Table 2). This can be explained by a positive, statistically significant correlation between D-dimers on the one hand and both WBC and HBP on the other (D-dimers : WBC: *r* = 0.4873, *p* < 0.05; D-dimers : HBP: *r* = 0.2631, *p* < 0.05).

**Figure 2:**
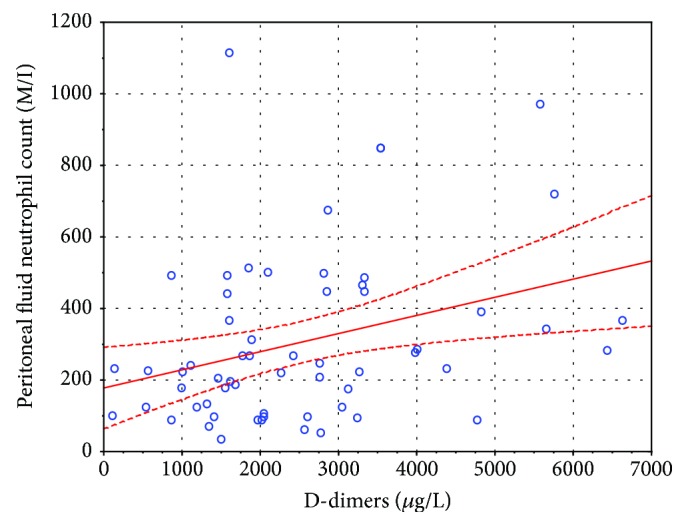
A scatterplot of D-dimers and the PMN count with four outliers removed—see text. The regression line is shown by a continuous line, and 95% confidence intervals are shown as a dashed line.

Figure 1 shows a weak, yet statistically significant, correlation between plasma D-dimers and the peritoneal fluid neutrophil count (*r* = 0.4783; *r*^2^ = 0.2287; *p* = 0.00007). The regression line is shown as a continuous line and 95% confidence intervals as dashed lines around the regression line. Note the four records of patients with D-dimers > 8000 *μ*g/L, which at the first glance seemed to be important influential points, but this possibility was ruled out by repeating the analysis without those points and obtaining a regression line with a similar slope which had an *r* coefficient that was still statistically significant (*r* = 0.3158; *r*^2^ = 0.0997; *p* = 0.0140; see Figure 2).

Unfortunately, none of the studied parameters allowed predicting the presence of SBP with a degree of certainty that would obviate the need for paracentesis in patients with ascites. Out of all the parameters studied, only D-dimers were independently correlated with the peritoneal fluid neutrophil count, and the correlation was weak to moderate, accounting for roughly 23% of variability found in the data (*r*^2^ = 0.2287).

A D-dimer cutoff value of 1500 ng/mL was determined optimal for ruling out SBP due to high sensitivity (0.968) and a high negative predictive value (0.929), although predictably, this marker was not useful for confirming SBP (for the aforementioned cutoff: specificity 0.406 and positive predictive value 0.612). However, only 22.2% of the studied patients had D-dimer levels below 1500 ng/mL. Other inflammatory markers (HBP, WBC, CRP, and PCT) showed little to no predictive value in this setting (see Tables 3–7).

## 5. Discussion

HBP can be an important piece of diagnostic information in patients with multiorgan failure [3]. We did not, however, find any study regarding the significance of HBP in the early diagnosis of SBP in the literature. Other biomarkers, such as CRP or PCT, are commonly known and have been thoroughly studied in patients with decompensated liver cirrhosis, ascites, and SBP. Some authors regard PCT and CRP in plasma as viable diagnostic tools for the diagnosis of SBP. PCT in particular seems to be regarded as more accurate [18, 19]. In a meta-analysis by Yang et al., PCT was found to be relatively sensitive and specific for SBP, although caution is advised as other causes of elevated PCT must be taken into consideration in the clinical setting [20].

In a study by Abdel-Razik et al., PCT, calprotectin, IL-6, and TNF-*α* concentrations in serum were higher in SBP patients than in those without SBP. A PCT level of 0.94 ng/mL showed high sensitivity (94.3%) and specificity (91.8%) for SBP. An important exclusion criterion in this study was antibiotic administration prior to the peritoneal tap. The authors of the study concluded that PCT and calprotectin both had a value in the evaluation in patients with suspected SBP [21]. Unlike in the study by Abdel-Razik et al., in our study, the PCT concentration was a poor predictor of SBP. One of the possible reasons for this is the fact that >30% of our patients was on antibiotic therapy prior to the peritoneal tap, whereas Abdel-Razik et al. had a more select sample. Our study suggests that PCT does not perform well as a marker of SBP in the heterogenous group of everyday clinical practice (see Table 7).

In a recent study by Mousa et al., a CRP level of 11.3 mg/dL had high sensitivity (88.9%) and specificity (92.6%) for SBP [22], yet in our study, CRP was a poor predictor of SBP (see Table 6).

Previous studies have shown the usefulness of D-dimers in the evaluation of patients with decompensated liver cirrhosis suspected of SBP and bacteraemia [23, 24]. Our study suggests that the diagnosis of SBP in patients with low D-dimers can be deemed unlikely, but further research with a larger sample size and possibly a more select sample of patients is needed to determine what the significance of this observation is in clinical practice.

## 6. Conclusions

Only the D-dimer levels were independently correlated with the ascitic fluid PMN count. D-dimers < 1500 ng/mL make the diagnosis of SBP unlikely (NPV = 92.9%), although the peritoneal tap is still the reference method in such situations.

Although both WBC and HBP correlate with the statistical significance with the ascitic fluid PMN count in the simple regression model, the strength of this correlation is negligible and is not statistically significant in the multiple linear correlation model. In our study, the determination of either HBP or other classic inflammatory markers (WBC, CRP, and PCT) was not of any diagnostic benefit in the diagnosis of SBP.

## Figures and Tables

**Table 1:** Baseline characteristics of the studied group.

Parameter	Median	Mean ± SD
Age (years)	60.0	61.3 ± 10.5
WBC (G/L)	5.6	6.3 ± 2.8
CRP (mg/L)	23.0	28.6 ± 19.7
PCT (ng/mL)	<0.05	0.4 ± 1.2
HBP (ng/mL)	63.4	93.5 ± 98.4
PLT (G/L)	120.0	149.1 ± 125.7
D-dimers (ng/mL)	2411.0	3187.0 ± 2851.0

**Table 2:** Correlation coefficient parameters (first column) and PMN for the simple and multiple linear regression models. Statistically significant coefficient *p* (with an alpha value of 0.05) in italics; *r*: Pearson's correlation coefficient; *r*^2^: Pearson's correlation coefficient squared; N/a: not applicable.

Parameter	Simple linear regression Pearson's correlation			Multiple linear regression *p*
	*r*	*r* ^2^	*p*	
Age (years)	−0.0195	0.0004	0.8796	>0.05
Sex/gender	N/a	N/a	N/a	>0.05
WBC (G/L)	0.2867	0.0822	*0.0227*	>0.05
CRP (mg/L)	0.1816	0.0330	0.1544	>0.05
PCT (ng/mL)	−0.0992	0.0098	0.4393	>0.05
HBP (ng/mL)	0.2915	0.0850	*0.0205*	>0.05
PLT (G/L)	0.0609	0.0037	0.6354	>0.05
D-dimers (ng/mL)	0.4783	0.2287	*0.00007*	*r* = 0.4783; *r*^2^ = 0.2287; *p* = 0.00007

**Table 3:** The sensitivities, specificities, positive predictive values (PPV), and negative predictive values (NPV) for D-dimers as a marker of spontaneous bacterial peritonitis.

D-dimer cutoff	Sensitivity	Specificity	PPV	NPV
≥1000 ng/mL	96.8%	18.7%	53.6%	85.7%
≥1250 ng/mL	96.8%	25.0%	55.6%	88.9%
≥1500 ng/mL	96.8%	40.6%	61.2%	92.9%
≥1750 ng/mL	83.9%	50.0%	61.9%	76.2%
≥2000 ng/mL	71.0%	53.1%	59.5%	65.4%

**Table 4:** The sensitivities, specificities, positive predictive values (PPV), and negative predictive values (NPV) for heparin-binding protein (HBP) as a marker of spontaneous bacterial peritonitis.

HBP cutoff	Sensitivity	Specificity	PPV	NPV
≥50 ng/mL	61.3%	46.9%	52.8%	55.6%
≥100 ng/mL	35.5%	75.0%	57.9%	54.5%
≥150 ng/mL	25.8%	90.6%	72.7%	55.8%
≥200 ng/mL	19.3%	90.6%	66.7%	53.7%
≥250 ng/mL	12.9%	93.7%	66.7%	52.6%

**Table 5:** The sensitivities, specificities, positive predictive values (PPV), and negative predictive values (NPV) for the peripheral blood leukocyte count (WBC) as a marker of spontaneous bacterial peritonitis.

WBC cutoff	Sensitivity	Specificity	PPV	NPV
≥6000/*μ*L	61.3%	71.9%	67.9%	65.7%
≥7000/*μ*L	41.9%	75.0%	61.9%	57.1%
≥8000/*μ*L	29.0%	90.6%	75.0%	56.9%
≥9000/*μ*L	22.6%	93.7%	77.8%	55.6%
≥10,000/*μ*L	12.9%	96.9%	80.0%	53.4%

**Table 6:** The sensitivities, specificities, positive predictive values (PPV), and negative predictive values (NPV) for C-reactive protein (CRP) as a marker of spontaneous bacterial peritonitis.

CRP cutoff	Sensitivity	Specificity	PPV	NPV
10.0 mg/L	93.5%	15.6%	51.8%	71.4%
20.0 mg/L	58.1%	53.1%	54.5%	56.7%
30.0 mg/L	41.9%	78.1%	65.0%	58.1%
40.0 mg/L	35.5%	81.2%	64.7%	56.5%
50.0 mg/L	22.6%	90.6%	70.0%	54.7%

**Table 7:** The sensitivities, specificities, positive predictive values (PPV), and negative predictive values (NPV) for procalcitonin (PCT) as a marker of spontaneous bacterial peritonitis.

PCT	Sensitivity	Specificity	PPV	NPV
0.10 ng/mL	41.9%	75.0%	61.9%	57.1%
0.25 ng/mL	22.6%	81.2%	53.8%	52.0%
0.50 ng/mL	19.3%	84.4%	54.5%	51.9%
1.00 ng/mL	3.2%	93.7%	33.3%	50.0%

## Data Availability

The data used to support the findings of this study are available from the corresponding author upon request.

## References

[1] Tapper H., Karlsson A., Morgelin M., Flodgaard H., Herwald H. (2002). Secretion of heparin-binding protein from human neutrophils is determined by its localization in azurophilic granules and secretory vesicles. *Blood*.

[2] Heinzelmann M., Mercer-Jones M. A., Flodgaard H., Miller F. N. (1998). Heparin-binding protein (CAP37) is internalized in monocytes and increases LPS-induced monocyte activation. *The Journal of Immunology*.

[3] Fisher J., Linder A. (2017). Heparin-binding protein: a key player in the pathophysiology of organ dysfunction in sepsis. *Journal of Internal Medicine*.

[4] Bentzer P., Fisher J., Kong H. J. (2016). Heparin-binding protein is important for vascular leak in sepsis. *Intensive Care Medicine Experimental*.

[5] Linder A., Christensson B., Herwald H., Björck L., Åkesson P. (2009). Heparin-binding protein: an early marker of circulatory failure in sepsis. *Clinical Infectious Diseases*.

[6] Tverring J., Vaara S. T., Fisher J. (2017). Heparin-binding protein (HBP) improves prediction of sepsis-related acute kidney injury. *Annals of Intensive Care*.

[7] Stjärne Aspelund A., Hammarström H., Inghammar M. (2017). Heparin-binding protein, lysozyme, and inflammatory cytokines in bronchoalveolar lavage fluid as diagnostic tools for pulmonary infection in lung transplanted patients. *American Journal of Transplantation*.

[8] Tranah T. H., Vijay G. K. M., Ryan J. M., Abeles R. D., Middleton P. K., Shawcross D. L. (2017). Dysfunctional neutrophil effector organelle mobilization and microbicidal protein release in alcohol-related cirrhosis. *American Journal of Physiology Gastrointestinal and Liver Physiology*.

[9] Caregaro L., Alberino F., Amodio P. (1998). Nutritional and prognostic significance of serum hypothyroxinemia in hospitalized patients with liver cirrhosis. *Journal of Hepatology*.

[10] Mølle I., Thulstrup A. M., Vilstrup H., Sørensen H. T. (2001). Increased risk and case fatality rate of pyogenic liver abscess in patients with liver cirrhosis: a nationwide study in Denmark. *Gut*.

[11] Thulstrup A. M., Sørensen H. T., Schønheyder H. C., Møller J. K., Tage‐Jensen U. (2000). Population-based study of the risk and short-term prognosis for bacteremia in patients with liver cirrhosis. *Clinical Infectious Diseases*.

[12] Englesbe M. J., Kubus J., Muhammad W. (2010). Portal vein thrombosis and survival in patients with cirrhosis. *Liver Transplantation*.

[13] Soresi M., Giannitrapani L., D'Antona F. (2006). Interleukin-6 and its soluble receptor in patients with liver cirrhosis and hepatocellular carcinoma. *World Journal of Gastroenterology*.

[14] Puoti M., Bonacini M., Spinetti A. (2001). Liver fibrosis progression is related to CD4 cell depletion in patients coinfected with hepatitis C virus and human immunodeficiency virus. *The Journal of Infectious Diseases*.

[15] Koulaouzidis A., Bhat S., Saeed A. A. (2009). Spontaneous bacterial peritonitis. *World Journal of Gastroenterology*.

[16] Sheikhbahaei S., Abdollahi A., Hafezi-Nejad N., Zare E. (2014). Patterns of antimicrobial resistance in the causative organisms of spontaneous bacterial peritonitis: a single centre, six-year experience of 1981 samples. *International Journal of Hepatology*.

[17] Fiore M., Maraolo A. E., Gentile I. (2017). Current concepts and future strategies in the antimicrobial therapy of emerging Gram-positive spontaneous bacterial peritonitis. *World Journal of Hepatology*.

[18] Yuan L. Y., Ke Z. Q., Wang M., Li Y. (2013). Procalcitonin and C-reactive protein in the diagnosis and prediction of spontaneous bacterial peritonitis associated with chronic severe hepatitis B. *Annals of Laboratory Medicine*.

[19] Wu H., Chen L., Sun Y., Meng C., Hou W. (2016). The role of serum procalcitonin and C-reactive protein levels in predicting spontaneous bacterial peritonitis in patients with advanced liver cirrhosis. *Pakistan Journal of Medical Sciences*.

[20] Yang Y., Li L., Qu C. (2015). Diagnostic accuracy of serum procalcitonin for spontaneous bacterial peritonitis due to end-stage liver disease: a meta-analysis. *Medicine*.

[21] Abdel-Razik A., Mousa N., Elhammady D. (2016). Ascitic fluid calprotectin and serum procalcitonin as accurate diagnostic markers for spontaneous bacterial peritonitis. *Gut Liver*.

[22] Mousa N., Besheer T., Abdel-Razik A. (2018). Can combined blood neutrophil to lymphocyte ratio and C-reactive protein be used for diagnosis of spontaneous bacterial peritonitis?. *British Journal of Biomedical Science*.

[23] Zhu D., Lu F. (2015). Clinical significance of plasma D-dimer in patients with liver cirrhosis complicated with spontaneous bacterial peritonitis. *Chinese Journal of Gastroenterology*.

[24] Schwameis M., Steiner M. M., Schoergenhofer C. (2015). D-dimer and histamine in early stage bacteremia: a prospective controlled cohort study. *European Journal of Internal Medicine*.

